# Addressing challenges to human health in the Anthropocene epoch—an overview of the findings of the Rockefeller/Lancet Commission on Planetary Health

**DOI:** 10.1186/s40985-016-0029-0

**Published:** 2016-10-04

**Authors:** Andy Haines

**Affiliations:** Departments of Social and Environmental Health Research and of Population Health, London School of Hygiene and Tropical Medicine, London, UK

**Keywords:** Planetary health, Climate change, Environmental change, Human health, Policy

## Abstract

The report of the Rockefeller Foundation/Lancet Commission on Planetary Health described how human health directly depends upon the environment. It takes a broad perspective not only acknowledging climate change as the most important global environmental threat to health but also recognizing other impacts, including dramatic loss of tropical forests, land degradation, loss of biodiversity, declining freshwater resources, ocean acidification, and over-exploitation of fisheries. All pose challenges to human health gains, leading to the concept of planetary health—that the human condition is tied to natural systems. The Planetary Health Commission report highlights several major concerns arising from environmental change including impacts on food availability and quality, increases in natural disasters and population displacement, and newly emerging diseases, e.g. from zoonotic infections. Three challenges emerge from the report: the first is imagination, or conceptual challenges—better metrics are needed to assess human progress within the context of environmental change; the second is a lack of relevant knowledge, requiring more research on the inter-linkages between environmental change and health and on the effectiveness of potential solutions; and the third is implementation of solutions, ensuring that the science is translated into policy and practice. There are many opportunities to promote planetary health including developing sustainable and healthy cities, encouraging more resilient health systems and disaster preparedness, reducing food waste, preserving ecosystems, and redirecting harmful subsidies in food, agriculture, fishery and energy sectors. Many current trends are driven by inequitable, inefficient, and unsustainable patterns of resource consumption and technological development, coupled with population growth, but solutions lie within reach. Prosperity must be redefined as an enhancement of the quality of life and the delivery of improved health for all, together with respect for natural systems.

## Main Text 

The report of the Rockefeller Foundation/Lancet Commission on Planetary Health [1] described how human health ultimately depends on the state of the natural systems. It is complementary to the work of the Lancet Commission on Climate Change [2] and takes a broader perspective on global environmental change, acknowledging that climate change is probably the single most important environmental change, but there are many others that can separately and in combination have wide ranging impacts on human health.

Human health has advanced tremendously in recent decades, for example, there has been an increase in life expectancy of over 20 years since the middle of the last century and a dramatic decline in childhood death rates, of over 70 %. But this has all come at a considerable environmental cost. Global average temperature has increased by 1 °C since pre-industrial times, and based on the commitments that were made in the run up to the COP21 Paris, the increase could amount to around 2.7 °C or more by the end of the century in absence of further actions. There are many other changes as well, including dramatic loss of tropical forests, one of the factors that is driving the loss of biodiversity that is occurring at rates 100-fold greater than pre-human times. Freshwater resources are in decline in many parts of the world and about three billion people live in locations that are subject to varying degrees of water stress, partly because of depletion of aquifers, which cannot be replenished in human lifetimes. Carbon dioxide is dissolving in the ocean leading to increasing acidification with probable major impacts on marine ecosystems. A single species, *Homo sapiens*, is now dominating the global environment, which has led an increasing number of scientists to call our epoch the Anthropocene, in recognition of the dominant role played by humanity. There are likely to be very major consequences for human health due to these changes, which are still incompletely understood. In particular, there are very few studies of the health implications of interactions between different environmental changes. One of the major concerns arising from the Planetary Health Commission report was the effect of multiple environmental changes on food availability and quality. Climate change itself will likely reduce crop yields, particularly in tropical and subtropical regions in the next few decades and probably in temperate regions in the second half of the century. Carbon dioxide fertilization is making some types of crops (C3 crops) grow a little faster, but it is also changing their nutrient quality, so it is reducing micronutrient levels. Declines in pollinators are occurring in many parts of the world, probably as a result of a number of environmental changes, with important implications for the yield of crops that depend on pollinators. A paper that appeared together with the Commission report suggested there could be an extra 1.4 million deaths a year if all the pollinators (such as bees) were lost largely because of the declines of fruit and vegetable availability, increasing the risk of non-communicable diseases and increasing infectious diseases because of reductions in vitamin A intake in some populations [3].

The report also outlined the potential effects of multiple environmental changes on disasters and displacement of populations. It gave the example of Pakistan, which is facing a combination of challenges: population growth, which is the highest outside Sub-Saharan Africa, and recent exposure to very large-scale floods and droughts affecting over 10 million people, displacing many people from their homes. And as the Commission report went to press, there was a major heatwave with temperatures over 42 °C in parts of southern Pakistan. There is already evidence that labourers in parts of Pakistan are beginning to move from rural to urban areas because they cannot work in the very intense summer heat that will only get worse [4].

Many emerging diseases are zoonotic infections that are often related to changes in agricultural practices and land use, increasing mixing of human populations with animal populations. In the Commission report, there was a case study of Ebola, which provides a dramatic example of how such outbreaks can disrupt fragmented and weak health systems. These challenges are also likely to get worse in the future.

The report identifies three types of challenges that need to be addressed. One is imagination, or conceptual challenges, for example, the tendency to focus on flawed indicators such as GDP growth as the main indicator of human progress. However, economic growth may be profoundly inequitable and associated with unsustainable environmental damage. Better metrics are needed for assessing human progress against the background of environmental change. The second challenge is that of a lack of knowledge and relevant information, which requires more research on the inter-linkages between environmental change and health and on the effectiveness of potential solutions. It is encouraging to see that two major research funders, the Rockefeller Foundation and the Wellcome Trust, have risen to the challenge of investing in research to address these planetary health issues. Forging better links between environmental data and human health data is essential to advance understanding, and Future Earth (see accompanying paper) provides an opportunity to do so. The third set of challenges is implementation challenges, which need to be addressed to make sure that the science gets into policy and practice. These require surmounting barriers such as those related to poor governance and vested interests as well as implementing policies to reform damaging subsidies and taxes.

There are a number of opportunities to promote planetary health, for example, by developing sustainable and healthy cities, including reducing greenhouse gas emissions from fossil fuel combustion with resulting improvements in fine particulate air pollution, and making cities more resilient against climate change. Green spaces can reduce the urban heat island effect, and they may also help to protect biodiversity and to promote mental health. Also, sustainable transport systems which promote public transportation and active travel—walking and cycling—can reduce air pollution and increase physical activity. Watershed conservation can help provide a clean water supply for cities, whilst reducing biodiversity loss, soil erosion, and flooding. Programmes to improve slums and informal housing can reduce vulnerability to disasters and temperature extremes, increase access to clean household energy, and help to address poverty.

More resilient health systems that can rebound from shocks stronger than before are essential to deliver a diverse range of services, which promote universal health coverage, and prepare for and respond to disasters. They will require much better disease surveillance systems that detect and control emerging diseases rapidly. Another example of a policy that contributes to improving planetary health is the reduction of food waste. About 30 % of the world’s total agricultural land is used to produce food that is never eaten and strategies to reduce food waste will need to address poor practices in harvesting, storage, transportation, marketing, and consumption. Many crops are not fed directly to humans but are used to feed animals because of the increasing demands for animal products. There are conversion inefficiencies which vary according to the type of animal product (being particularly high for beef), and also many animal products are associated with higher greenhouse gas emissions compared with vegetables, particularly from ruminants because they produce methane in their intestines. Increasing fruit and vegetable consumption and reducing animal product consumption in high-consuming populations hold the potential to reduce environmental impacts and improve health. This will be a crucial area for research and the disciplinary silos between health, agricultural, and environmental scientists must be overcome so they can work together.

Ecosystem strategies can help to increase disaster resilience, for example, preserving wetlands and mangroves can help protect coastal populations against tidal waves and sea-level rise, and coral reefs can provide a safe haven for many fish on which human populations depend. Around 90 % of the world’s fisheries are currently fully exploited or overexploited, and over two billion people depend on fisheries for a significant proportion of their protein intake. Around 70 % of aquaculture depends on supplemental feeds, and there is widespread use of antibiotics and pesticides. More sustainable aquaculture is needed in order to take the pressure off natural fisheries. There is also increasing evidence that forest conservation can protect biodiversity and health as well as reducing greenhouse gas emissions. For example, 300,000 people a year die from air pollution caused by landscape fires, in part to clear forests and peatlands for commercial use. This is particularly striking in parts of Southeast Asia.

The Commission also showed that there are many subsidies in the food, agriculture, fisheries, and energy sectors that are driving humanity in the wrong direction. They are allowing us to exploit resources, which are in turn causing serious damage to the environment and human health. A recent International Monetary Fund report has shown, for example, that there are annual energy subsidies of around $5 trillion. Some of them are direct but most of them are caused by the fact that we do not pay the full economic costs of air pollution and of climate change. Policies should be enacted to reverse damaging subsidies and also to reform tax systems to ensure that taxes reflect the damaging externalities of economic activities, for example, by implementing carbon taxes to reduce greenhouse gas emissions.

The Sustainable Development Goals (SDGs) will be a major driver of policies worldwide over the next 15 years. Planetary health can act as an integrating framework across the SDGs. Health is only reflected directly in goal 3, but many other goals address key determinants of health, for example, goal 1 on poverty, goal 2 on sustainable agriculture and nutrition, goal 6 on water and sanitation, goal 7 on access to clean energy, goal 11 on sustainable clean cities, and others on preserving biodiversity in terrestrial and marine ecosystems. The goals, targets, and indicators for the SDGs reflect many of the key dimensions of planetary health.

In conclusion, despite the many challenges, solutions lie within reach. They should be based on the redefinition of prosperity to focus away solely from the growth of GDP towards the enhancement of the quality of life and the delivery of improved health for all, together with respect for the integrity of natural systems.

## Abbreviations

SDGs: Sustainable Development Goals
